# The Physicochemical Characterization of Unconventional Starches and Flours Used in Asia

**DOI:** 10.3390/foods9020182

**Published:** 2020-02-12

**Authors:** Ng C. F. Grace, Christiani Jeyakumar Henry

**Affiliations:** 1 Clinical Nutrition Research Centre (CNRC), Agency for Science, Technology and Research (A*STAR), 14 Medical Drive, Singapore 117599, Singapore; jeya_henry@sics.a-star.edu.sg; 2 Department of Biochemistry, Yong Loo Lin School of Medicine, National University of Singapore, 8 Medical Drive, Singapore 117596, Singapore

**Keywords:** tapioca, sweet potato, sago, water chestnut, high amylose maize, red rice, kithul, starch, flour, physicochemical properties

## Abstract

Starches and flours used commonly in Asia (tapioca, sweet potato, sago, water chestnut, and high amylose maize starch, red rice and kithul flour) were characterized in terms of their chemical composition, morphological, functional, pasting, thermal, gelling and in vitro digestibility properties. It was observed that the differences in their chemical composition and structure influenced their properties. High amylose maize was the most stable, thus it required the highest gelatinization temperature which was observed in both the differential scanning calorimetry (DSC) and pasting profiles. Kithul flour had a significantly lower rate of digestion (*p* < 0.05) than the other samples (except for high amylose maize starch). Unlike high amylose maize starch, it had a gelatinization temperature that could be achieved during cooking, and had good gelling properties.

## 1. Introduction

Starch is the main carbohydrate in foods and is an important food ingredient for many food products. Most of the research have focused on the mainstream starches such as maize, potato, rice, cassava and wheat starches, which are the major sources used for the commercial extraction of starch globally [1]. In recent years, there has been greater interest in the study of starch and flours from less conventional sources. 

There is a growing demand for starchy foods with low glycemic index (GI), which are more slowly digested and metabolized, leading to a more gradual and smaller rise in blood glucose. Much work has been done on mainstream starches in a bid to alter the structure to influence the digestibility of the starch. However, many of the unconventional starches and flours commonly used in Asia such as sweet potato (*Ipomoea batatas)*, sago (*Metroxylon sagu*), water chestnut *(Eleocharis dulcis*) starches, red rice (*Oryza rufipogon)* and kithul flour (*Caryota urens*), have not been extensively characterized. Starch is extracted from the roots of the sweet potato, and the corm of the water chestnut. Sago and kithul are from the palm family and extraction from the pith of the stem yields the flour. These starches/flours are available commercially in native state but not much research has been done in terms of systematically characterizing them and studying their functional properties. Some characterization work of these starches/ flours have been carried out comparing between species after starch isolation [2,3,4], or in comparison with wheat flour or rice starch [5]. However, there is no paper that has done comprehensive physicochemical characterization looking at the structural, morphological, crystallinity, thermal, rheological and gelation properties using the same analytical instruments and parameters in a way that allows meaningful comparison of the starches/flours of interest in this paper. In addition, the properties that have been reported in different papers cannot be compared, owing to the differences such as the concentration used, the storage time and the methods of measurement. The present study aimed to look at the physicochemical characterization of unconventional starches and flours that are available commercially but not well characterized.

## 2. Materials and Methods 

### 2.1. Materials

The starch and flour samples used in this study were commercial samples: tapioca starch (Ng Nam Bee Marketing, Singapore), sweet potato starch (White Swallow Brand, Singapore), sago starch (Yiak Say Hang Food Industries, Singapore), water chestnut starch (Pan Tang Brand, Guangzhou, China), high amylose maize starch (Ingredion, Singapore), red rice flour (Ruhunu Foods, Pallekale, Sri Lanka) and kithul flour (Sri Lanka). Tapioca (*Manihot esculenta*) starch is selected as a basis of comparison against these starches to evaluate the functional properties with a mainstream commercial starch commonly used in Asia. High amylose maize starch is selected as it is a commercial food ingredient that is used in low GI food products. All samples were sieved through a 250 µm sieve to remove impurities, and sealed in bags for future analysis. The abbreviations for the samples are in brackets: high amylose maize starch (HAMS), kithul flour (KF), red rice flour (RRF), sago starch (SGS), sweet potato starch (SPS), tapioca starch (TS) and water chestnut starch (WCS). 

### 2.2. Chemical Composition

The chemical composition of the starches and flours were quantitatively determined by an accredited chemical laboratory (SETSCO Services Pte Ltd, Singapore) using the following methodology: Moisture (AOAC 950.46), protein (AOAC 981.10, 991.20), fat (AOAC 996.06), ash (AOAC 930.30), dietary fiber (AOAC 985.29), available carbohydrates by difference (FAO 1998) and phosphorus by inductively coupled plasma-optical emission spectrometry (ICP-OES) after microwave digestion [6].

The total starch and amylose content of the starches and flours were determined using the megazyme amylose/amylopectin assay procedure, ultilizing the commercial kit (Megazyme Ireland International Ltd, Bray, Ireland).

### 2.3. Morphological Properties 

The granule shapes and “maltese cross” were viewed under a light microscope (BX53F2, Olympus, Tokyo, Japan) equipped with a CCD camera, with and without a polarizing plate, according to the method used by Cai and Wei [7]. Starch and flour suspensions (1%) were prepared, and a drop of suspension was placed on the microscope slide and covered with a cover slip. 

The granule size distribution was examined by a laser diffraction particle size analyzer (PSA1190, Anton Paar, GmbH, Graz, Austria). Starch granules were fully suspended in water and an obscuration of 15% ± 1% was achieved for all measurements. The volume-weighted mean diameter (µm), D [3,4], was reported as an average of three replicates. 

The crystalline patterns of starches were performed using an X-ray diffractometer (D8 Discover micro XRD, Bruker, Billerica, MA, USA) equipped with a two-dimensional detector Vantec 500 and micro focus Cu source operated at 50 kV, 800 µA and 40 watt. The diffraction patterns were obtained from a scanning angle range between 3° and 30° (2θ) at a step size of 0.02°. 

### 2.4. Functional Properties

Swelling power of starches were determined using a modified method by Leach et al. [8]. Starch suspensions (2%, w/w db) were heated in a water bath at 95 °C for 30 min. The slurry was then cooled and centrifuged (Rotina 380, Hettich Lab, Tuttlingen, Germany) at 3000 g for 20 min. The supernatant was evaporated in an air oven at 105 °C for 3 h. The dried residue from the supernatant and sediment were weighed. The swelling power (SP) and water solubility index (WSI) were calculated using Equations (1) and (2). The mean is obtained from three replicates:

(1) $$\mathrm{SP}(g/g)\;=\;\frac{weight\;of\;sediment}{weight\;of\;dry\;sample\;\times \;(1\;-\;WSI)},$$

(2) $$\mathrm{WSI}(g/g)\;=\;\frac{Total\;solids\;in\;superntant}{weight\;of\;dry\;sample}.$$

Freeze thaw stability was determined by the method of Hoover and Ratnayake [9]. Starch suspensions (6%, w/w db) were heated in a water bath at 95 °C for 30 min with constant stirring. The slurry was poured into separate tubes and were subject to cold storage at 4 °C for 16 h and then frozen at −16 °C for 24 h. To measure freeze thaw stability, the frozen gels were thawed at 25 °C for 6 h and then refrozen at −16 °C. Five cycles of freeze thaw were performed. The tubes were centrifuged at 1000 *g* for 20 min and the released water (%) was measured as freeze thaw stability.

### 2.5. Pasting Properties

The pasting profile of the starch suspensions (10% w/w, dry weight basis) were obtained using the starch analyzer (MCR 302, Anton Paar, Graz, Austria). A total weight of 20 g starch suspension was placed in the starch cell and dispersed at a vane speed of 960 rpm, which reduced to 160 rpm after 1 min. The heating and cooling cycle was programmed according to the following: with the exception of high amylose maize starch, samples were held at 50 °C for 1 min, heated to 95 °C at a rate of 2 °C/min, maintained at 95 °C for 5 min, cooled to 50 °C and held for 2 min. A pressure cell system (Anton Paar, Graz, Austria) is required for high amylose maize starch to heat the starch above 100 °C in order for gelatinization to occur. High amylose maize starch was held at 50 °C for 1 min, heated to 150 °C at a rate of 2 °C/min, maintained at 150 °C for 5 min, cooled to 50 °C and held for 2 min. The parameters including pasting temperature, peak viscosity, trough viscosity (minimum viscosity at 95/150 °C), final viscosity (viscosity at 50 °C), breakdown value (peak viscosity–trough viscosity) and setback value (final viscosity–trough viscosity) were recorded. Duplicate pasting curves were measured. 

### 2.6. Thermal Properties 

The thermal properties of the starches were measured using a differential scanning calorimeter (DSC 204 F1, NETZSCH, Selb, Germany) according to Lan et al. [10]. Three mg of starch and 10 Μl of water was weighed in an aluminum pan, hermetically sealed and equilibrated overnight at room temperature. An empty sealed pan served as the reference. The scanning temperature range was between 20–120 °C and at a heating rate of 2 °C/min, respectively. Onset (T_o_), peak (T_p_) and conclusion (T_c_) temperatures (°C), and enthalpy change (J/g) were determined. Proteus® software (NETZSCH, Selb, Germany) was used to calculate the enthalpy of the endothermic peak. The onset (T_o_), peak (T_p_), and completed (T_c_) temperatures of the gelatinization peak were determined from the intersection of tangents fitted to the leading and trailing flanks of the peak with the baseline. 

### 2.7. Textural Properties of Gel

Gel texture properties were analyzed using methods by Wang et al. [11] and Kaur et al. [12] with slight modification. Starch/flour suspensions (10% w/w in water) were heated in water bath at 95 °C for 30 min. The gelatinized flour and starch paste was poured into a plastic container (Ø = 30 mm) and kept at 20 ± 2 °C for 24 h to form a solid gel. The gels were unmoulded from the containers and double compression was performed on the samples at room temperature using a texture analyzer, TA-Xtplus (Stable Micro Systems Ltd, Surrey, UK). Each gel sample was compressed to a strain of 50% with a cylindrical probe (Ø = 75 mm) at a speed of 1 mm/s with a recovery time of 10 s between the first and second compressions. From the force–time curve, the following parameters were obtained [13]: the maximum force (N) of the first compression was recorded as the hardness of the gel; the ratio of the gel’s detected height in the second compression to the original compression distance was recorded as springiness, the ratio of the area of work of the second compression to that of the first compression was recorded as cohesiveness; and the multiplication of the hardness, cohesiveness and springiness values were recorded as the chewiness.

### 2.8. In Vitro Enzymatic Digestibility

An in vitro digestion model that mimics human gastrointestional digestion [14,15,16,17] was used to evaluate the digestibility of the starch and flour samples. Starch/flour suspensions (25% w/w in water) were heated in a water bath at 95 °C for 30 min and allowed to set overnight at 20 °C. Gel samples (2.5 g), which were in distilled water (30 Ml), were digested in containers placed in a circulating water bath at 37 °C. The samples were constantly stirred at 200 rpm. 

Oral phase: After stirring for 30 min, a solution of 0.1 Ml of 10% α-amylase (≥10 units/mg solid) dissolved in distilled water was added and stirred for 1 min. A total of 0.8Ml of 1 M HCL was added to adjust the Ph to Ph 2.5 (±0.2), using a Ph meter (SevenCompact S220™, Mettler Toledo®, Columbus, OH, USA).

Gastric phase: 1 Ml of 10% pepsin (≥250 units/mg solid) dissolved in 0.05 M HCl was added. Gastric digestion was concluded after 30 min of stirring at 37 °C. For neutralizing the gastric phase HCl, 2 Ml of 1 M NaHCO3 and 5 Ml of 0.2 M maleate buffer (Ph 6) were added into the mixture. Distilled water was added to the mixture to a final volume of 55 Ml and stirred continuously at 150 rpm until the temperature of the mixture reached 37 °C.

Pancreatic phase: 0.1 Ml of amyloglucosidase (≥260 U/Ml, aqueous solution) and 1 Ml of 5% pancreatin in 0.2 M maleate buffer was added and stirred continuously.

A total of 0.5 Ml aliquots was taken at baseline (before start of oral phase), at the end of oral and gastric phase, and at 20, 60, 90, 120 and 180 min from when the pancreatic phase started. To stop the enzymatic digestion, the drawn aliquots were added to 2 Ml of ethanol. The ethanolic digesta samples were stored at 4 °C for the analysis of reducing sugar.

The analysis of reducing sugars in the ethanolic digesta samples was measured by a dinitrosalicylic acid (DNS) colourimetric method [16,18]. The samples were centrifuged at 1000 *g* for 10 min. 0.5 Ml aliquots of the supernatant were drawn and added to 0.25 Ml of 1% amyloglucosidase (≥260 U/Ml, aqueous solution) dissolved in 0.1 M acetate buffer (Ph 5.2). The mixture was vortexed and incubated at 37 °C for 10 min. Then, 0.75Ml of DNS mixture was then added. This was prepared by mixing 0.5 mg/Ml of glucose, 4 M NaOH and DNS reagent in a ratio of 1:1:5. After the mixture was heated for 15 min at 95 °C, 4 Ml of distilled water was added to each sample and cooled for 10 min. Absorbance was measured at 530 nm by a UV-VIS spectrophotometer (UV-2600, Shimadzu, Kyoto, Japan). The blank and standard was distilled water and glucose (10 mg/Ml), respectively. The results of the glucose concentration were presented as mg/g starch.

### 2.9. Statistical Analysis

The data reported in all the tables were mean values and standard deviations of triplicate observations unless otherwise stated. There were four replicates for both the gel texture analysis and the in vitro digestion. The comparisons between samples for the texture analysis and in vitro digestibility were analyzed by a one-way ANOVA with Tukey’s multiple comparison test (*p* < 0.05) using GraphPad Prism version 8.0.0 for Windows, GraphPad Software (San Diego, CA, USA). 

## 3. Results and discussion

### 3.1. Chemical Composition 

The chemical composition of the various starch and flours is listed in Table 1. Sago, sweet potato and tapioca starches had trace amount of protein while red rice flour had the highest amount. The trace amount of fat in kithul flour, sago and sweet potato starches indicates that these have been defatted. The high protein, fat, ash and dietary fiber content is attributed to the raw state of the red rice flour. The degradation during starch extraction resulted in lower values of protein, fat and ash. High amylose maize and water chestnut starches have higher starch content than the amount of available carbohydrates. This is attributed to their resistant starch content which has been reported for high amylose maize starch [19]. 

### 3.2. Morphological Properties

**Figure 1:**
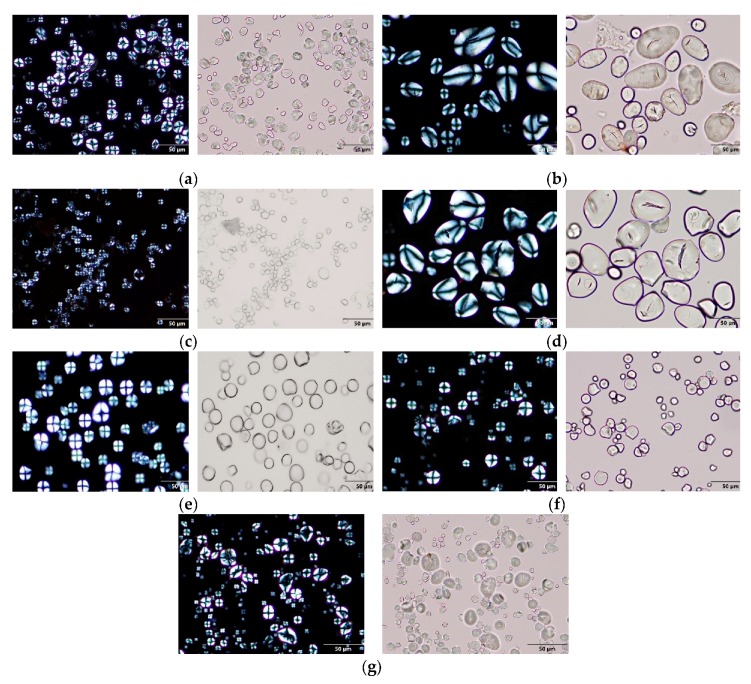
The microscope images of (**a**) HAMS, (**b**) KF, (**c**) RRF, (**d**) SGS, (**e**) SPS, (**f**) TS, and (**g**) WCS under normal and polarized light (40× objective). HAMS—high amylose maize starch, KF—kithul flour, RRF—red rice flour, SGS—sago starch, SPS—sweet potato starch, TS—tapioca starch and WCS—water chestnut starch.

Particle size distributions and crystallinity of the starch and flour samples are presented in Table 2. Among the samples, granules of kithul and sago were the largest (>25 µm), followed by granules of sweet potato, water chestnut, high amylose maize and tapioca (10–25 µm), and red rice was smallest (5–10 µm). Both kithul and sago are starches from the pith of the palm tree. Kithul, red rice, sago, sweet potato and water chestnut was of type A crystallinity, high amylose maize was of type B crystallinity and tapioca starch was of type C crystallinity. Crystallinity type was in agreement with previous findings [20,21,22,23]. Figure 1 shows the microscope images of the starch and flour granules under normal and polarized light. Kithul and sago granules are a mixture of oval and truncated shape, while the rest of the starch granules were a mixture of round, elliptical and polygonal shapes. 

### 3.3. Functional and Pasting Properties 

Table 3 shows the water solubility index, swelling power and freeze thaw stability of the starch and flour gels. Sago had the highest swelling power which explains its high peak viscosity just before the granules rupture. The higher protein and fat content in red rice flour and water chestnut starch restricted their swelling capacity [24]. The double helices structure of amylose is more stable and heat is needed to break the hydrogen bonds so that the amylose can bond with water molecules which contributes to the swelling of the starch granule [25]. Thus, the high amylose content in high amylose maize starch restricted its swelling capacity. The freeze thaw stability of red rice flour, tapioca and water chestnut starches were the poorest as the water released (%) was the highest among the samples.

**Figure 2:**
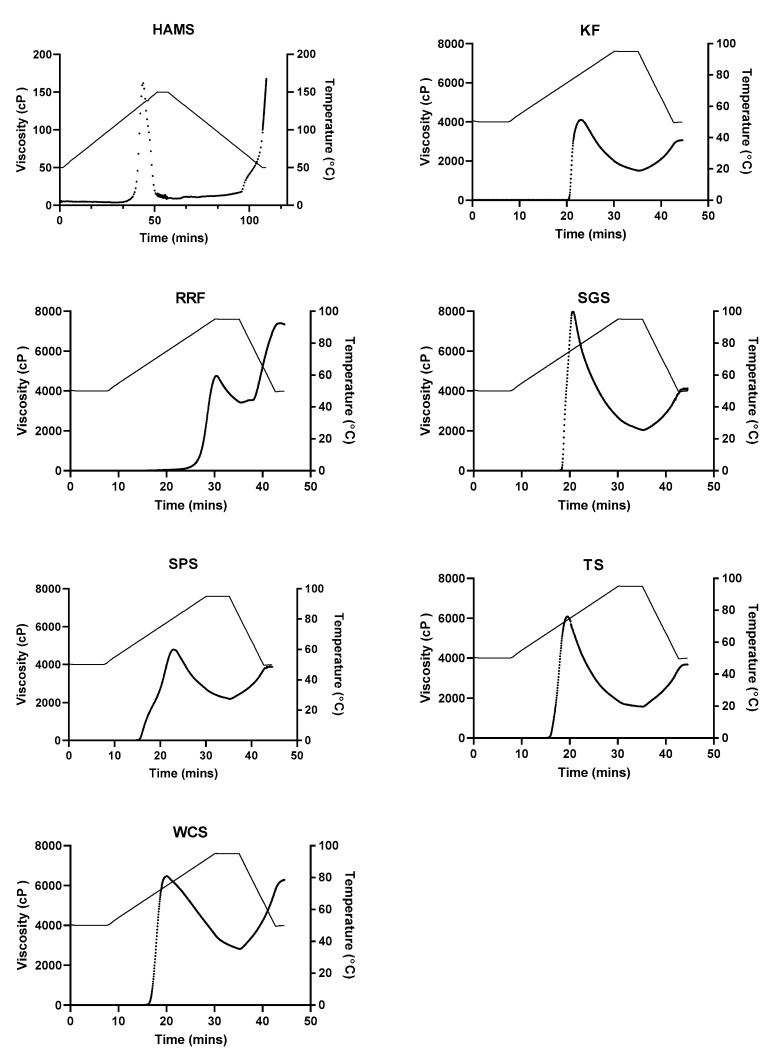
The pasting profiles of 10% w/w starch or flour suspensions. The viscosity curve is denoted by ●, plotted on the left axis; while the temperature curve is denoted by ꟷ, plotted on the right axis. HAMS—high amylose maize starch, KF—kithul flour, RRF—red rice flour, SGS—sago starch, SPS—sweet potato starch, TS—tapioca starch and WCS—water chestnut starch.

Figure 2 shows the pasting profiles of all the starches and flours. High amylose maize starch had very high amylose contents which restricted the swelling and resulted in no increase in viscosity at 95 °C. Table 4 shows the pasting properties of the 10% w/w starch or flour suspension. Since amylopectin is responsible for the swelling in starch granules, having a high amylose content delayed the onset of gelatinization [26,27], attributing to the higher pasting temperatures observed in high amylose maize starch, sago starch, kithul flour and red rice flour. Additionally, the amylose–lipid complexes formed could have contributed to the higher pasting temperatures in high amylose maize starch and red rice flour. The lower peak viscosities observed in the kithul and red rice flour may be due to the lower amount of starch content present. Sago starch had the highest swelling power, which resulted in the highest peak viscosity due to the large swollen granules. However, upon shear and further heating, the sago starch had the highest breakdown viscosity, which indicated that sago is susceptible to high viscosity loss and may not be suitable in food applications that involve high shear during processing. The higher breakdown observed may be due to the larger frictional forces between the swollen granules. Water chestnut starch, tapioca starch and red rice flour had high set-back viscosities which agrees with the results of the freeze thaw stability test which is also an indicator of the tendency of starch to retrograde [28]. 

### 3.4. Thermal Properties

Table 5 shows the thermal properties of the starches and flours. The high amylose maize starch had high gelatinization onset temperature due to the high amylose content, which agreed with the results presented by other authors [29]. The amylose double helices present require high amount of energy to be disrupted, which explains the high gelatinization temperature [30]. There was a second peak observed in the gelatinization DSC curve for high amylose maize starch which was due to the amylose–lipid complex (data not shown). Kithul flour, sago and sweet potato starches have higher gelatinization conclusion temperatures which is attributed to their larger granule size. The high pasting temperatures of kithul and sago starch is in agreement with their high peak gelatinization temperature. Larger granules have poorer hydration efficiency and swelling capacity as compared to smaller granules [31]. Granule size had been reported to have a direct relationship with enthalpy [32,33] which is reflected in the higher enthalpy for kithul and sago and a smaller enthalpy for red rice flour. A possible reason for red rice flour to have a smaller enthalpy than tapioca starch could be attributed to the fact that red rice flour had a larger proportion of small starch granules than tapioca starch. The D_10_, D_50_ and D_90_ (number based distribution) for red rice flour and tapioca starch was 0.22, 0.35, 0.60 µm and 0.31, 0.44, 1.60 µm, respectively. 

### 3.5. Textural Properties of Gel

The hardness, springiness, cohesiveness and chewiness values of the 10% w/w starch and flour gels with the exception of high amylose maize starch, are shown in Table 6. The hardness of the gels were in the following sequence: kithul flour > water chestnut starch > sago starch > sweet potato starch > tapioca starch > red rice flour. The hardness of the gels was significantly different from each other except for sweet potato starch which was not significantly different from sago and tapioca starch. All gels were similar in terms of springiness except for red rice flour which was noticeably more brittle as some of the gels fractured during the first compression. The gels from kithul flour and water chestnut starch were significantly chewier than the rest, while the gels from sago, sweet potato and tapioca starches had similar chewiness. The gel from red rice flour was not chewy at all as it fractured upon the first compression, indicating poor elastic properties. 

### 3.6. In Vitro Digestibility

**Figure 3:**
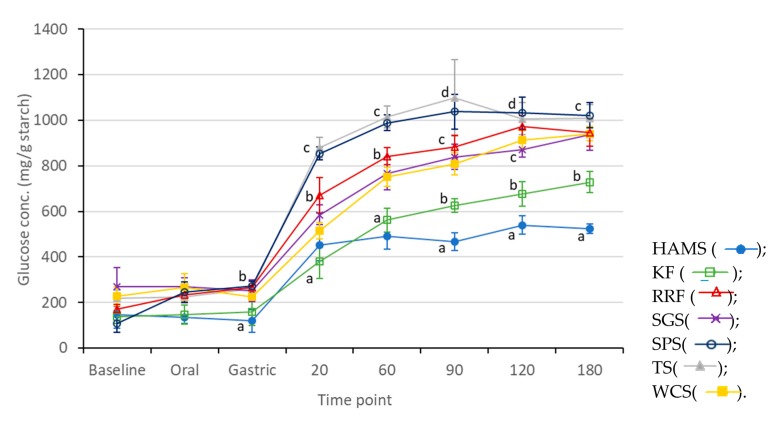
The in vitro digestibility of the starch/flour gels. The amount of sugar released across the digestion phases was expressed as means ± standard deviations plotted in error bars (*n* = 4). Values with different letters (a–d) in the same time point are significantly different (*p* < 0.05) according to Tukey’s test. HAMS—high amylose maize starch, KF—kithul flour, RRF—red rice flour, SGS—sago starch, SPS—sweet potato starch, TS—tapioca starch and WCS—water chestnut starch.

Figure 3 shows the amount of sugar released across the digestion phases of the starch and flour gels. The high amylose maize starch had the lowest amount of free sugars released at the end of 180 min as it had the highest amount of dietary fiber. In addition, both the gels of kithul flour and high amylose maize starch had significantly lower rates of digestibility as compared to the other gels at 20 min of the pancreatic phase. This may be explained by the higher amounts of protein, amylose and dietary fiber present in them. Although the red rice flour had a high amount of protein, amylose and dietary fiber, it is suggested that its small granules result in a faster rate of digestion [34,35] as compared to kithul and high amylose maize. A combination of factors such as the smaller granule size, lower amounts of protein, fat, dietary fiber and amylose resulted in the higher rates of digestibility observed in the tapioca and sweet potato starch gels. 

## 4. Conclusions 

Starches and flours that are non-mainstream but commonly used in Asia were studied. The chemical composition and structure of the starches varied, which resulted in the significant differences in the in vitro digestibility, functional, thermal, pasting and textural properties. From this study, it was discovered that kithul flour had a significantly lower rate of digestion than the other samples (except for high amylose maize starch), had a gelatinization temperature that could be achieved during cooking and had good gelling properties. It would be interesting to have a greater understanding of its chemical composition such as how the amylopectin fine structure may influence its properties. Future research on unconventional sources of starch from the tropical region enable us to secure starches with unusual rheological, sensory, nutritional or organoleptic properties. 

## Figures and Tables

**Table 1:** The chemical composition of the starch and flour (data are means ± standard deviations).

Starch/Flour	Moisture (%)	Protein (%)	Fat (%)	Ash (%)	Dietary Fiber (%)	Available Carbohydrates (%)	Phosphorus (mg/100 g)	Starch (%)	Amylose (%)
**HAMS**	9.17 ± 0.08	0.75 ± 0.00	0.87 ± 0.07	0.04 ± 0.01	39.41 ± 0.2	49.78 ± 0.10	23.20 ± 0.14	94.84 ± 0.08	59.74 ± 2.82
**KF**	10.22 ± 0.21	0.57 ± 0.04	<0.01 ± 0.00	0.17 ± 0.00	1.79 ± 0.36	87.24 ± 0.25	21.61 ± 0.13	55.89 ± 16.5	26.16 ± 0.32
**RRF**	5.78 ± 0.28	9.94 ± 0.00	1.26 ± 0.10	0.60 ± 0.03	2.73 ± 0.35	79.69 ± 0.31	17.21 ± 0.10	41.82 ± 5.85	26.10 ± 2.07
**SGS**	9.31 ± 0.39	<0.10 ± 0.00	<0.01 ± 0.00	<0.01± 0.00	0.53 ± 0.01	90.05 ± 0.39	9.84 ± 0.06	89.56 ± 0.60	26.98 ± 1.15
**SPS**	11.16 ± 0.09	0.10 ± 0.00	<0.01 ± 0.00	0.10 ± 0.01	0.81 ± 0.01	87.83 ± 0.10	13.73 ± 0.08	84.02 ± 1.04	21.36 ± 0.60
**TS**	9.97 ± 0.10	<0.10 ± 0.00	0.19 ± 0.01	0.02 ± 0.00	0.68 ± 0.00	89.04 ± 0.10	7.86 ± 0.05	88.80 ± 0.13	24.14 ± 0.12
**WCS**	10.51 ± 0.23	0.36 ± 0.05	0.49 ± 0.04	0.28 ± 0.01	0.67 ± 0.02	87.70 ± 0.29	11.86 ± 0.07	90.00 ± 2.50	22.45 ± 0.51

HAMS—high amylose maize starch, KF—kithul flour, RRF—red rice flour, SGS—sago starch, SPS—sweet potato starch, TS—tapioca starch and WCS—water chestnut starch.

**Table 2:** The morphological properties of the starch and flours (data are means ± standard deviations).

Starch/Flour	Crystallinity	D_4,3_ (µm)	D_10_ (µm)	D_50_ (µm)	D_90_ (µm)
**HAMS**	B	15.53 ± 1.14	6.24 ± 0.20	12.83 ± 0.61	26.53 ± 1.14
**KF**	A	45.50 ± 0.59	18.66 ± 0.10	38.95 ± 0.25	75.40 ± 1.24
**RRF**	A	24.37 ± 0.48	5.41 ± 0.05	16.32 ± 0.57	51.87 ± 2.55
**SGS**	A	35.77 ± 0.10	17.71 ± 0.05	31.39 ± 0.08	54.67 ± 0.16
**SPS**	A	22.36 ± 1.13	9.35 ± 0.20	18.97 ± 0.75	37.06 ± 2.32
**TS**	C	15.56 ± 0.01	7.33 ± 0.01	13.74 ± 0.01	24.44 ± 0.01
**WCS**	A	20.70 ± 0.05	8.08 ± 0.03	16.91 ± 0.02	35.51 ± 0.08

D_4,3_ is the volume-weighted mean diameter; D_10_, D_50_ and D_90_ are the granule diameter at which 10%, 50% and 90% of all the granules by volume are smaller, respectively. HAMS—high amylose maize starch, KF—kithul flour, RRF—red rice flour, SGS—sago starch, SPS—sweet potato starch, TS—tapioca starch and WCS—water chestnut starch.

**Table 3:** The functional properties of the starch and flours (data are means ± standard deviations).

Starch/Flour	WSI (g/100 g)	SP (g/g)	Freeze Thaw Stability (%)
**HAMS**	4.78 ± 0.84	2.55 ± 0.06	-
**KF**	18.50 ± 1.73	31.58 ± 0.56	24.91 ± 2.04
**RRF**	17.38 ± 0.79	12.11 ± 0.54	43.87 ± 3.60
**SGS**	12.20 ± 3.31	38.15 ± 3.08	28.43 ± 3.83
**SPS**	26.85 ± 6.38	32.12 ± 3.07	29.36 ± 1.83
**TS**	18.82 ± 7.32	32.10 ± 6.78	48.14 ± 4.33
**WCS**	13.28 ± 1.40	20.75 ± 0.62	38.38 ± 3.26

WSI and SP are the water solubility index and swelling power of a 2% w/w starch suspension after being heated at 95 °C, respectively. HAMS—high amylose maize starch, KF—kithul flour, RRF—red rice flour, SGS—sago starch, SPS—sweet potato starch, TS—tapioca starch and WCS—water chestnut starch.

**Table 4:** The pasting properties of 10% w/w starch or flour suspension.

Starch/Flour	PT (°C)	PV (cP)	HV (cP)	BV (cP)	FV (cP)	SV (cP)
**HAMS**	123.3	161.8	8.6	153.2	166.2	157.6
**KF**	75.0	4105.0	1501.0	2604.0	3061.5	1560.5
**RRF**	66.2	4763.0	3422.5	1340.5	7342.0	3919.5
**SGS**	70.2	8025.0	2038.5	5986.5	4127.5	2089.0
**SPS**	63.5	4799.0	2195.5	2603.5	3876.5	1681.0
**TS**	63.6	6097.5	1573.0	4524.5	3679.5	2106.5
**WCS**	65.5	6472.0	2820.0	3652.5	6287.0	3467.0

PT—pasting temperature; PV—peak viscosity; HV—hot paste viscosity; BV—breakdown viscosity (PV-HV); FV—final viscosity; SV—setback viscosity (FV-HV). HAMS—high amylose maize starch, KF— kithul flour, RRF—red rice flour, SGS—sago starch, SPS—sweet potato starch, TS—tapioca starch and WCS—water chestnut starch.

**Table 5:** The thermal properties of the starch and flours (data are means ± standard deviations).

Starch/Flour	T_o_ (°C)	T_p_ (°C)	T_c_ (°C)	Enthalpy (J/g)
**HAMS**	91.9 ± 2.5	110.7 ± 0.2	132.4 ± 9.8	3.1 ± 0.6
**KF**	74.3 ± 0.4	77.4 ± 0.5	81.5 ± 0.6	4.2 ± 0.5
**RRF**	60.7 ± 0.2	66.2 ± 0.1	71.1 ± 1.2	1.6 ± 0.1
**SGS**	61.9 ± 0.4	72.5 ± 0.2	81.1 ± 0.7	6.1 ± 0.9
**SPS**	61.8 ± 0.4	66.8 ± 0.0	79.3 ± 0.7	3.0 ± 0.2
**TS**	61.4 ± 0.5	68.1 ± 0.1	76.6 ± 0.8	3.1 ± 0.2
**WCS**	60.2 ± 0.8	68.1 ± 0.6	74.4 ± 0.7	3.6 ± 0.2

T_o_, T_p_ and T_c_ are the onset, peak and completed temperatures of the gelatinization peak, respectively. HAMS—high amylose maize starch, KF—kithul flour, RRF—red rice flour, SGS—sago starch, SPS—sweet potato starch, TS—tapioca starch and WCS—water chestnut starch.

**Table 6:** The textural properties of the starch and flour gels (10% w/w in water). Values with the same alphabet superscript (a–e) in the same row are not significantly different.

Starch/Flour	KF	RRF	SGS	SPS	TS	WCS
**Hardness (N)**	12.09 ± 0.62 ^a^	1.45 ± 0.08 ^b^	5.41 ± 0.41 ^c^	4.35 ± 0.34 ^c,d^	3.84 ± 0.29 ^d^	8.75 ± 0.91 ^e^
**Springiness**	0.92 ± 0.02 ^a^	0.52 ± 0.02 ^b^	0.89 ± 0.01 ^a^	0.94 ± 0.02 ^a^	0.90 ± 0.01 ^a^	1.29 ± 0.37 ^a^
**Cohesiveness**	0.82 ± 0.01 ^b^	0.26 ± 0.01 ^c^	0.75 ± 0.02 ^b^	0.73 ± 0.02 ^b^	0.73 ± 0.02 ^b^	0.90 ± 0.02 ^a^
**Chewiness**	9.10 ± 0.51 ^a^	0.19 ± 0.02 ^b^	3.64 ± 0.36 ^c^	3.01 ± 0.35 ^c^	2.54 ± 0.19 ^c^	9.98 ± 2.18 ^a^

HAMS—high amylose maize starch, KF—kithul flour, RRF—red rice flour, SGS—sago starch, SPS—sweet potato starch, TS—tapioca starch and WCS—water chestnut starch.
